# Microbially induced stabilized rammed earth: Compressive strength-capillary water absorption Co-relationship

**DOI:** 10.1016/j.heliyon.2025.e42680

**Published:** 2025-02-13

**Authors:** Neha Vivek A, Prasanna Kumar P, Divijendranatha Reddy

**Affiliations:** a Department of Civil Engineering, B.M.S. College of Engineering, Bengaluru, India; b Department of Biotechnology, B.M.S. College of Engineering, Bengaluru, India

**Keywords:** Rammed earth, Bacteria, Microbially induced calcium carbonate precipitation, Capillary water absorption

## Abstract

Rammed Earth (RE) is finding more applications as a sustainable construction material with cement as stabilizer. However, RE is susceptible to water absorption through pores, weakening the bond between the soil particles, reducing its strength and lifespan. Increased cement content, though enhances strength, does not effectively fill pores. There are no proven methods to fill pores with cementitious materials in structures like RE. In this study, Microbially Induced Calcium Carbonate Precipitation (MICP) has been adopted during casting to improve the compressive strength of cement-stabilized RE. MICP technique not only enhances the strength but also fills pores, inhibiting capillary water absorption thereby extending the lifespan of RE. The results of tests on MICP-induced specimen indicated that the compressive strength increased by an average of 26 %. The capillary water absorption reduced considerably with the bacterial induction. A relationship between compressive strength and capillary water absorption of RE is arrived. While further study is necessary for microbial stabilization to be used in RE, these preliminary data show promising results.

## 1 Introduction

It is well known that conventional building materials like concrete, produce a large amount of greenhouse gases. Hence, it has become crucial that alternative building materials and techniques need to be explored, which can be effective in the reduction of greenhouse gas emissions. Rammed Earth [1,2] is considered as one of the most energy-efficient building techniques using soil, sand and clay compacted in successive layers between rigid formwork. Modern RE construction uses cement as a stabilizer to improve its engineering properties and has been successfully adopted worldwide. The use of stabilizers such as cement has been widely observed for the past few years as it is easily accessible and provides the required strength characteristics. Cement content in the range of 6 %–10 % provides the required strength, but not the durability of RE. It is established from earlier research studies that a cement content of 6 % or higher provides higher compressive strength, while cement content greater than 15 % is considered as lean concrete and is not recommended for sustainable construction [3,4]. To attain higher strengths, it is necessary to create bonds with clay fraction of RE that do not disintegrate in moisture along with the inert matter of rammed earth [5]. However, various issues associated with Cement Stabilized Rammed Earth (CSRE) need further investigations and solutions. The loss of strength due to water absorption in the pores of RE is one such issue that affects its durability and structural integrity. In recent years, biomineralization by Microbially Induced Calcite Precipitation has been used as a bio cementing procedure in hardened concrete for improving strength and durability parameters [[6], [7], [8], [9], [10]]. MICP is also being used in soil stabilization, especially in sandy soils, where a bio-cement product is formed which enhances the properties of the soil [[11], [12], [13], [14], [15]]. MICP is primarily known for its use in soil stabilization, but its application in RE is relatively new. Majority of researchers have established the positive effect of employing MICP on cohesionless soils due to its high permeability, while improvement in soil using bacteria for cohesive soils is poorly understood. The process of bio-cementation increases the compressive strength of soils by producing soil binding compounds such as carbonates, silicates, hydroxides, phosphates and sulphates. Some research has been made on applying MICP to reduce the moisture absorption in bricks and compressed earth blocks [16,17]. The compressive strength of recycled brick is enhanced in the range of 5 %–20 % due to the refinement effect of CaCO_3_ precipitation [18]. MICP is a biological process, which occurs when microorganisms, a bacterium in general, leads the precipitation of calcium carbonate (CaCO_3_) crystals with the provision of an appropriate cementing solution [19]. The precipitation of calcium carbonate by bacteria via urea hydrolysis is a commonly used mechanism since it is most productive, controllable and energy-efficient [20]. Bacteria play a crucial role in producing the necessary enzymes like urease and carbonic anhydrate which favour the precipitation of CaCO_3_. The cementing solution should contain a calcium source and urea solution. Calcium chloride having high calcite yield and high urease activity is considered as a good calcium source for the MICP process [21]. A study on compacted granitic soil treated with MICP has shown that the calcium carbonate content increases (in the range of 6 %–10 %) with an increase in curing period leading to an increase in strength over longer periods [22]. The calcite precipitation on the surface and carbonate formation in the soil reduced the void ratio in the range of 5 %–15 % in MICP treated soils [23].

Urease positive bacteria is commonly used in the MICP process that have the ability to catalyse urea hydrolysis by supporting calcite production. Most frequently found urease positive bacteria belong to the genera of *Bacillus*. In a majority of the earlier studies on soil stabilization, researchers have used *Bacillus megaterium*, *Bacillus sphaericus* and *Bacillus pasteurii*, since they are found in soil and adapt well to the soil conditions. The compressive strength of intermediate compressible clay soil and low compressible clay soil induced with *Bacillus* bacteria resulted in a higher strength than that of soils treated with *Lysinibacillus* bacteria due to the high urease producing capability of *Bacillus* bacteria [13]. The quantity of cementing solution also plays a critical role in the precipitation of calcium carbonate. Excess calcium chloride reduces urease activity during the MICP process. It was concluded by previous researchers that the concentration of cementing solutions (CaCl_2_-urea) in the range of 10–250 mM gives higher compressive strength, beyond which the compressive strength of soil reduces [24].

Field application of this research findings may also be made with the use of powdered bacteria [11,25] achieved by Lyophilization [26]. MICP using calcium carbonate precipitation from cultivated and lyophilized *Bacillus pasteurii* bacteria, was found to be effective in reducing swelling potential, increasing stiffness and improving shear strength of expansive clays and sandy silts [25]. Although, lyophilized bacteria offer convenience, it is susceptible to loss of bacteria viability during the lyophilization process [27]. However, MICP by bacteria isolates creates stable mineral formations within the material by harnessing the natural processes of bacteria and enhancing its strength and resilience over time [28].

The aim of this research investigation is (i) to reduce the capillary water absorption and porosity and (ii) to enhance the compressive strength and properties of rammed earth. The study introduces the application of MICP for improving the mechanical properties and durability of rammed earth (RE) constructions, which is a novel approach. In this study, bacteria was induced in RE during the casting process and the stress-strain characteristics of RE after the MICP process were investigated on both the unstabilized and cement stabilized RE samples after 28 and 90 day curing periods. Further, the deposition of calcium carbonate crystals in the RE specimen was depicted by Scanning Electron Microscopy (SEM) and Energy Dispersive X-Ray Spectrum (EDX). The development of microbiologically-induced calcium carbonate as a stabilizer may offer an economical and environmentally safe alternative.

## 2 Materials and methods

### 2.1 Soil, manufactured sand and cement

**Fig. 1:**
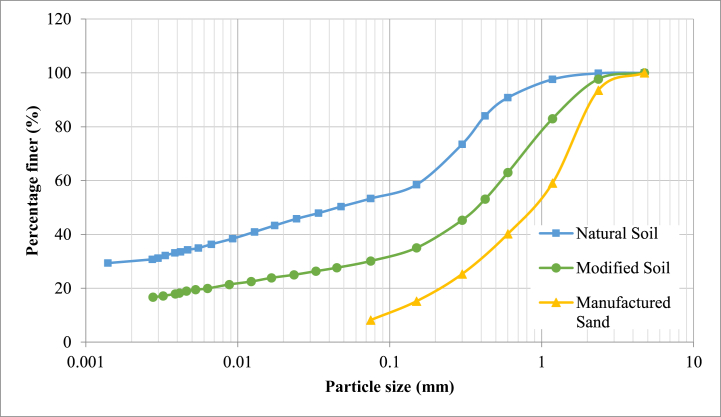
Grain size distribution curve of natural soil, modified soil and manufactured sand [35].

The soil utilised in this study was classified as clayey sand (SC) according to Unified Soil Classification System (USCS) [29,30] and had a clay content of 30 %, which was reconstituted to a clay content of 15 % by mixing an equal amount of manufactured sand (M-sand) in the ratio of 1:1 by weight since earlier studies have suggested that the optimum clay content for maximum compressive strength of RE is in the range of 13 %–16 % [5,31,32]. The M-sand used in this study belongs to Zone II of Indian Standards IS:383 [33]. The grain size distribution curve of natural soil and reconstituted soil is shown in Fig. 1. Table 1 gives the characteristics of natural soil and reconstituted soil. Ordinary Portland Cement (OPC) complying with Indian Standards IS-12269 [34] was used as a stabilizer for casting the rammed earth specimen.

**Table 1:** Properties of natural and modified soil [35].

Properties	Natural Soil	Modified Soil
1. Textural composition		
Sand (4.75–0.075 mm) (%)	52	69
Silt (0.075–0.002 mm) (%)	18	16
Clay (<0.002 mm) (%)	30	15
2. Atterberg's limits		
Liquid limit (%)	40	28
Plasticity index	17	12
3. Unified soil classification system (USCS)	SC	SC
4. Predominant clay mineral	Kaolinite	Kaolinite
5. Compaction characteristics		
Maximum dry density (kN/m^3^)	17.1	19.65
Optimum moisture content (%)	15	10.8
6. Specific gravity	2.66	

### 2.2 Microorganisms and growth conditions

To study and compare the effect of microbial stabilization of RE, three types of microorganisms from the *Bacillus* genera were employed. The urease-producing isolates *Bacillus megaterium* (NCIM 5472), *Bacillus sphaericus* (NCIM 2478) and *Bacillus* sp. (NCIM 2477) were procured from the National Collection of Industrial Microorganisms (NCIM), Pune, India. These bacteria are gram-positive and gram staining test on the bacterial cells confirmed positive results with a purple colour appearance. The media used for this study was Nutrient Broth, purchased from HiMedia, India. The cementation solution used for the investigation was 2 % urea solution (filter sterilized) and 25 mM CaCl_2._ They were added to nutrient broth-urea (NBU media) and the final pH of the NBU media was maintained at 8.0. The bacteria culture was incubated at 37 °C under shaking conditions at 120 rpm for 120 h in a mechanical shaking incubator. The growth profile of the bacteria was determined by the absorbance values, i.e., Optical Density (OD) is determined at 600 nm, which is termed as OD_600_. The OD_600_ is measured at regular intervals of time and the corresponding cells/ml were determined after incubation at 37 °C. The bacterial cells per mL (Y) were determined using equations (1), (2), (3)), where X is the value of OD_600_ [8,11,36]:

(1) $$Y=8.59\times 10^{7}X^{1.3627}\;for\;Bacillus\;sp\text{.}$$

(2) $$Y=3.25\times 10^{7}X^{1.990}\;for\;Bacillus\;megaterium$$

(3) $$Y=4.25\times 10^{7}X^{1.857}\;for\;Bacillus\;sphaericus$$

### 2.3 Urease activity

One unit of urease is described as the amount of enzyme hydrolysing 1μ mol of urea per minute [17]. The urease activity of all three bacteria was found by the phenol-hypochlorite assay method with 24-h intervals up to 120 h [37]. The standard used for the urease test was 1000 μM of ammonium chloride. A mixture containing 1 ml of 0.1 M potassium phosphate buffer (pH 8.0) and 2.5 ml of 0.1M urea was added to 250 μl bacterial culture filtrates. After incubation of the mixture at 37 °C for 5 min, 1 ml each of phenol nitroprusside and alkaline hypochlorite were added to the mixture and were incubated at 37 °C for 25 min. Optical Density (OD) was measured at 626 nm using a spectrophotometer and the amount of urease produced was determined.

### 2.4 Casting of RE specimens

As a part of this investigation, both unstabilized and cement-stabilized rammed earth samples were prepared to study the effect of microbially-induced stabilization. The Optimum Moisture Content (OMC) and Maximum Dry Density (MDD) of modified soil was 10.8 % and 19.7 kN/m^3^ respectively, determined by Standard Proctor tests. The OMC value adopted for casting of all RE specimens was 10.8 %, since there was only a marginal change after the addition of cement. The OMC in bacteria induced specimen also did not vary since the bacteria are fully grown and does not require additional moisture. The specimen were compacted to three densities of 18 kN/m^3^, 19 kN/m^3^ and 20 kN/m^3^.

A cylindrical mould with split halves having internal dimensions of 100 mm diameter and 300 mm height was used to produce a compacted cylindrical RE specimen of 100 mm diameter and 200 mm height maintaining an aspect ratio of 2. A rammer with a circular head was used to compact the RE cylinders. The RE specimen were compacted in three equal layers to obtain the required density. In order to have proper bonding between the layers of soil being compacted, an indenter with an oval-shaped pointed end was used to make indents between layers in addition to scratching with a sharp-edged tool.

Natural soil and manufactured sand (M-sand) were sieved through 4.75 mm sieve. The natural soil was modified by adding an equal amount of M-sand in the ratio of 1:1. For casting Cement Stabilized Rammed Earth (CSRE), cement at 8 % [3,4] of the weight of the dry mix of RE was mixed thoroughly into the modified soil. Water corresponding to the OMC was sprayed into this mixture and was mixed manually to obtain a uniform mix. For microbial calcification, bacterial cells were grown in NBU media until peak growth (OD_600_ = 1) was obtained. NBU media containing bacterial cells corresponding to OMC were added to the dry mix. A known quantity of prepared wet mix was equally parted into three divisions by weight. Each part was then filled into the mould and compacted into three layers of equal depth till the required density was achieved. After demoulding, microbial RE specimen and control specimen (without bacterial induction) were kept at room temperature of 25 °C to 27 °C for 24 h. All the specimen were cured with wet burlap for a period of 28 day. In order to evaluate the long-term strength characteristics of RE after bacterial induction, curing was continued for 90 day.

### 2.5 CaCO_3_ precipitation

The CaCO_3_ precipitate in the culture solution was collected after a period of 4 weeks. The bacterial culture was grown in 100 ml nutrient broth-urea media. The CaCO_3_ precipitate in the conical flask was filtered, washed with distilled water and dried in the oven for 48 h. The collected crystals were dried and quantified accurately using a digital balance with a least count of 0.001 g.

After testing the RE samples for compressive strength, 5 g of soil sample was mixed meticulously with 20 ml of 1 Molar hydrochloric acid (HCl). Hydrochloric acid dissolves the soluble calcium carbonate present in the soil. The mixture was then washed on filter paper with distilled water to remove all the soluble calcium from the soil particles. The soil particles remaining on the filter paper was oven dried and weighed. The difference in weight between the original soil sample (M_1_) and washed sample (M_2_) gives the total weight of calcium carbonate. The calcium carbonate content (CCC) is determined as:

(4) $$\text{CCC}\left(\%\right)=100-\left(\frac{M_{2}}{M_{1}}\right)\times 100$$

### 2.6 Compression test

**Fig. 2:**
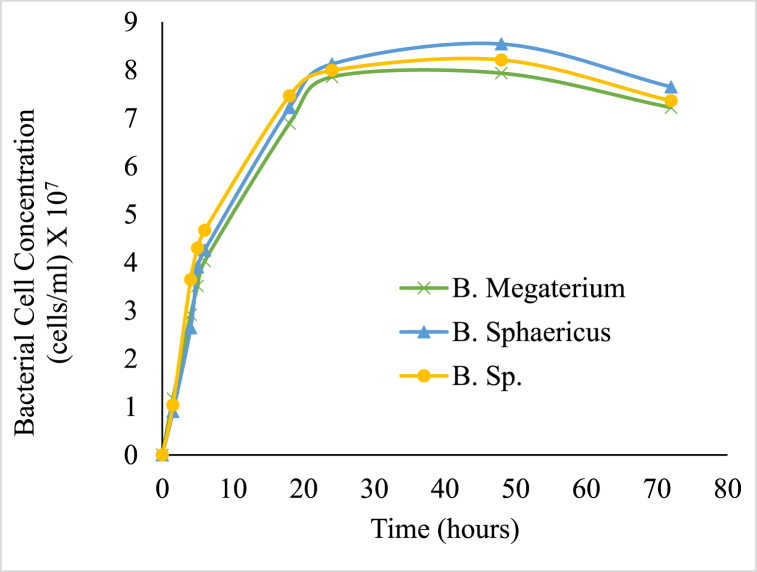
Bacterial Growth Profile with respect to time.

To evaluate the strength of RE samples, compression test after 28 and 90 day curing periods were conducted in a Universal Testing Machine (UTM) with a maximum loading capacity of 1000 kN (Fig. 2). Cylinders for compressive strength were capped with gypsum plaster before testing to provide a uniform surface for testing. The specimen was saturated in a water bath after capping and the saturated specimens were tested for compressive strength. The axial deformation was measured with a demountable mechanical strain gauge having a least count of 0.002 mm. The rate of loading was set at 2 N/mm^2^/min till failure. The initial tangent modulus and strain at peak stress were also determined from the compressive strength tests.

### 2.7 Capillary water absorption test

The capillary water absorption coefficient of the specimen was determined as per ASTM C1585 [38]. RE specimen of dimension 100 mm diameter and 50 mm height were prepared according to specifications outlined in ASTM C1585 [38]. The specimen were cured under wet burlap for 28 day and then oven dried to maintain a constant dry weight prior to the water absorption test. The sides of the oven-dried specimen were sealed with a hydrophobic gel and the top surface was covered with a plastic sheet and secured with an elastic band. The top and sides of the specimen were sealed to ensure that water absorption occurred exclusively through the bottom surface, since the seal prevents evaporation and from external factors from influencing the results, thus providing accurate data on capillary absorption properties.

Two rods of 6 mm diameter as supports were placed at the bottom of a pan and the pan was filled with water so that the water level was 3 mm above the top of the rods. A steady water level for consistent capillary rise within the specimen was maintained.

**Fig. 3:**
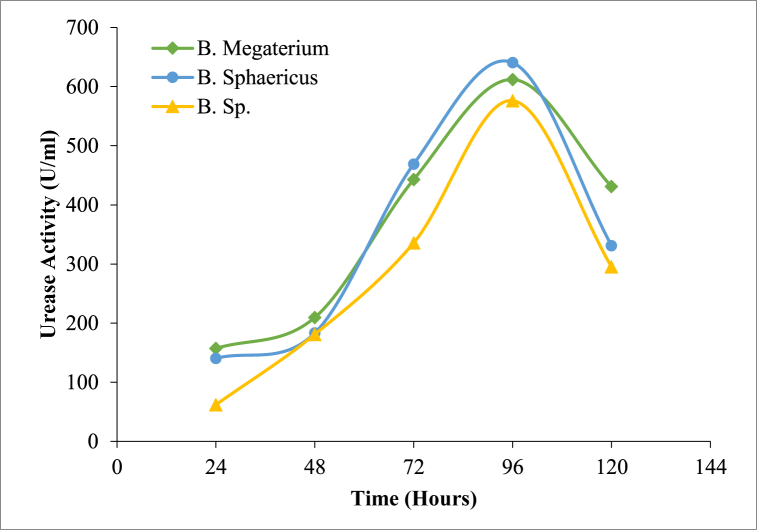
Urease Activity with respect to time.

Water absorption was recorded at regular intervals of 5, 10, 20, 30 and 60 min and thereafter every hour up to 6 h. After the elapse of initial 6 h, weight of the specimen was recorded once in 24 h till the weight remained constant. The additional water from the surface of the RE specimen was cleaned with a blotting paper before measuring its weight. The specimen in capillary water absorption test is shown in Fig. 3.

### 2.8 Microscopic analysis

The soil sample after testing for compressive strength was powdered and sieved through a 75-μm sieve for Scanning Electron Microscope (SEM) analysis. The sample was mixed and kept overnight in a 2 % glutaraldehyde solution and was washed with 0.1M sodium phosphate buffer solution for approximately 20 min. Further, it was dehydrated in 50 %, 70 %, 85 %, and 95 % ethanol solution for 10 min and kept in 100 % ethanol solution until the sample dried. The samples were dehydrated in increasing concentrations of ethanol solution to prevent mechanical damage to the intercellular structures [39]. The morphology of the powdered sample was analysed using a VEGA3 TESCAN electron microscope with a provision of an Energy Dispersive X-ray spectrum (EDX).

## 3 Results and discussions

### 3.1 Growth profile and urease activity

The bacterial growth profile study indicated that the optimum bacterial cell concentration for all three species of bacteria was similar and followed the same growth profile. The growth profile was studied up to 72 h for all the three species. A pH of 8 and a temperature of 37 °C was adopted during urease activity and growth conditions. The bacterial cells per mL were determined using equations (1), (2), (3). Optical density for higher urea hydrolysis activity was in the range of OD_600_ = 0.8 to 1.2, which is in accordance with the previous studies [40]. It was observed that the bacteria could grow to an optimum concentration and utilize all the nutrients present in the nutrient broth (NB). The growth curve was similar for all three *Bacillus* genera and no significant difference was observed (Fig. 2). The peak growth period for the bacteria was observed at 48 h (OD_600_ = 1.0). The highest cell concentration determined was 8.54 × 10^7^ cells/mL at a pH of 10 after 48 h. The pH of the media increased as the bacterial growth increased. This rise in pH may be the reason for the calcium carbonate precipitation, which occurs during the hydrolysis of urea [41].

Urease production by all the three *Bacillus* increased considerably with time and the maximum urease produced was logged on the 4th day at 640 U/ml (Fig. 3). After 96 h, a sharp decline in the activity of the urease was observed. The rate of urease activity indicated that the calcium carbonate precipitation accelerates from the consequent increase of pH in neighbouring media due to the existence of ammonia ions during enzymatic urea hydrolysis [42]. Hence, an active contribution of urease is the fundamental part of biochemical calcium carbonate precipitation. The formation of carbonate ions from bicarbonate ions present in water depends strongly on the pH and, hence, under alkaline conditions, carbonate ion concentration increases. Therefore, in alkaline environments with substantial amounts of calcium ions and carbonate ions, calcium carbonate precipitation readily occurs [43].

### 3.2 Stress-strain relationship of RE specimens at 28 day curing period

**Fig. 4:**
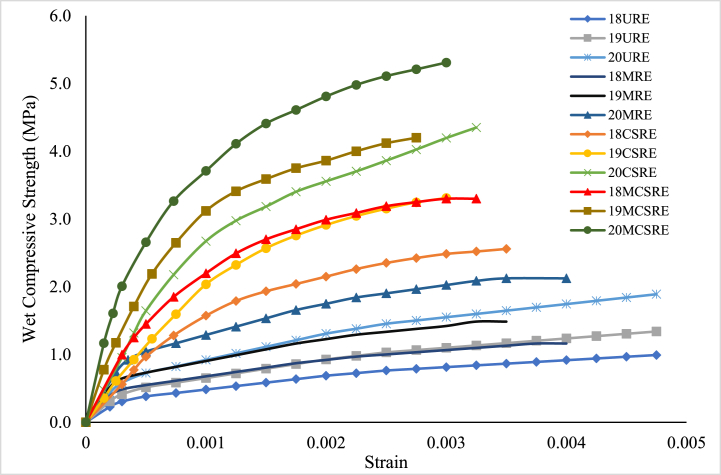
Stress-Strain Curve for URE, MRE, CSRE and MCSRE specimen at 28 day curing.

The stress-strain curves for unstabilized rammed earth (URE) (modified soil), cement stabilized rammed earth (CSRE), microbially induced rammed earth (MRE) and microbially induced cement stabilized rammed earth (MCSRE) of three densities were obtained after the wet compressive tests at 28 day curing periods and are illustrated in Fig. 4. From Fig. 4, it is seen that the stress-strain curves for all rammed earth specimens are non-linear. The average moisture content was observed to be 11 % at the time of testing. It is evident that with an increase in density, the wet compressive strength also increases [5,32]. The average compressive strength of URE and CSRE specimens increased by 35 % when density increased from 18 kN/m^3^ to 19 kN/m^3^ and the same increment was observed when the density increased from 19 kN/m^3^ to 20 kN/m^3^. This increase in strength may be because of the reduction in pores leading to an effective bond among the particles of RE [32]. However, for RE specimens induced with bacteria, when the density increased from 18 kN/m^3^ to 19 kN/m^3^, the increase in wet compressive strength was 27 % with respect to CSRE. The increase in strength when the density increased from 19 kN/m^3^ to 20 kN/m^3^ was 24 %.

A significant increase in strength was observed when RE was stabilized with cement. The wet compressive strength of URE varied in the range of 1.0 MPa–1.89 MPa for all three density variations of 18 kN/m^3^, 19 kN/m^3^ and 20 kN/m^3^. When RE was stabilized with cement the strength increased significantly to the range of 2.56 MPa–4.35 MPa for the same density variations. The average compressive strength of RE increased by 2.5 times after cement stabilization. With the presence of cement in the soil matrix, there is a sufficient quantity of cementitious material available which forms bonds between the soil particles leading to higher strength of CSRE which was also observed by other researchers [3,4,32].

**Fig. 5:**
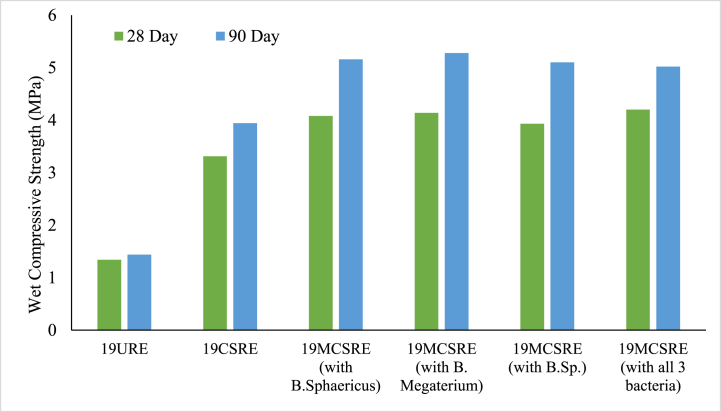
Wet Compressive Strength of microbially stabilized RE for a density of 19 kN/m.^3^.

The wet compressive strength of CSRE with only cementing solution (NBU media without bacteria isolates) was determined before the addition of bacteria. The wet compressive strength increment of CSRE with only cementing solution was at an average of 9 % when compared to URE and it was 2.79 MPa for 18 kN/m^3^, 3.64 MPa for 19 kN/m^3^ and 4.68 MPa for 20 kN/m^3^. This increase might be due to the stimulation of bacteria species already present in the soil [44]. The compressive strength after the induction of bacteria varied marginally amongst the three bacterial species, *Bacillus megaterium*, *Bacillus sphaericus* and *Bacillus* sp. for all the three densities, 18, 19 and 20 kN/m^3^. The average strength values were 3.17 MPa, 4.05 MPa and 5.27 MPa respectively for the three densities (Fig. 5). It was observed that, highest compressive strength was obtained for microbial-induced cement stabilized RE (MCSRE) when compared with all other RE specimen. An average of 26 % improvement in compressive strength was noted in microbially induced CSRE. The average coefficient of variation of wet compressive strength was 8 %.

From the results obtained, it was analysed that a substantial improvement in compressive strength at 28 day curing for MCSRE specimens might be credited to the behaviour of bacterial cells in the RE matrix. Initially, bacterial cells obtained abundant nutrients, but the growth might have been low because of new environmental conditions for the microbes. This was evident by the low precipitation of calcium carbonate during the initial days [45]. A high pH value of 10 was observed during the initial days. The cells might be in an inactive condition and as days progressed, the cells might have started to grow gradually. With an increase in cell growth, calcium carbonate crystals precipitated on the outside of cells as well as inside the MCSRE matrix leading to an increase in compressive strength because of the filling of voids in the RE matrix [21,46]. With a further growth in cells, the supply of nutrients and oxygen to the cells decreases, which leads to either conversion of cells into endospores or death of cells that behave as organic fibres aiding in the increase of compressive strength of MCSRE [7,47]. Hence, it may be deduced that the increase in compressive strength of MCSRE at 28 day is primarily because of the filling of voids inside the MCSRE specimen with the MICP process.

The Initial Tangent Modulus (ITM) and the strain at ultimate compressive strength were established from the stress-strain characteristics and the results are tabulated in Table 2. The 28 day strain at peak stress is lower for cement stabilized specimen indicating that it has turned relatively brittle when compared with unstabilized RE. The strain at peak stress for URE was within the range of 0.0045–00048 and for MRE it was within the range of 0.0035–0.0040. The peak strain for CSRE was within the range of 0.003–00035 while for MCSRE it was within the range of 0.0028–0.0033. These results indicate that the unstabilized specimens (URE and MRE) were relatively ductile with higher strain values but lower strength values. However, the specimens stabilized with cement and bacteria (CSRE and MCSRE), were more brittle with lower strains but with higher strength.

**Table 2:** Stress-Strain Characteristics of RE specimens at 28 day curing period.

Dry Density (kN/m^3^)	URE		MRE		CSRE		MCSRE	
	ITM (MPa)	Strain at peak stress	ITM (MPa)	Strain at peak stress	ITM (MPa)	Strain at peak stress	ITM (MPa)	Strain at peak stress
18	980	0.0048	1500	0.0040	1600	0.0035	2680	0.0033
19	1320	0.0045	2070	0.0035	2450	0.0030	4130	0.0028
20	1670	0.0045	2550	0.0040	3290	0.0033	5490	0.0030

The ITM values increased by two times with an increase in density from 18 kN/m^3^ to 20 kN/m^3^ for all stabilized RE specimen. The ITM of specimen stabilized with cement was greater than that of specimen without cement stabilization by about 1.5 times. The ITM of URE was within the range of 980 MPa–1670 MPa. The ITM of CSRE was within the range of 1500 MPa–2550 MPa while that of MCSRE, it was within the range of 2680 MPa–5490 MPa. The increment in ITM of MCSRE was 1.6 times when compared with CSRE and 3.1 times that of the control specimen. These outcomes show that the modulus of cement stabilized RE increases significantly after microbial induction and is sensitive to variations in density. From these observations, it can be analysed that with microbial stabilization the voids in stabilized RE specimens are largely filled leading to an increase in strength which consequently increases the ITM.

### 3.3 Stress-strain relationship of RE specimens at 90 day curing period

**Fig. 6:**
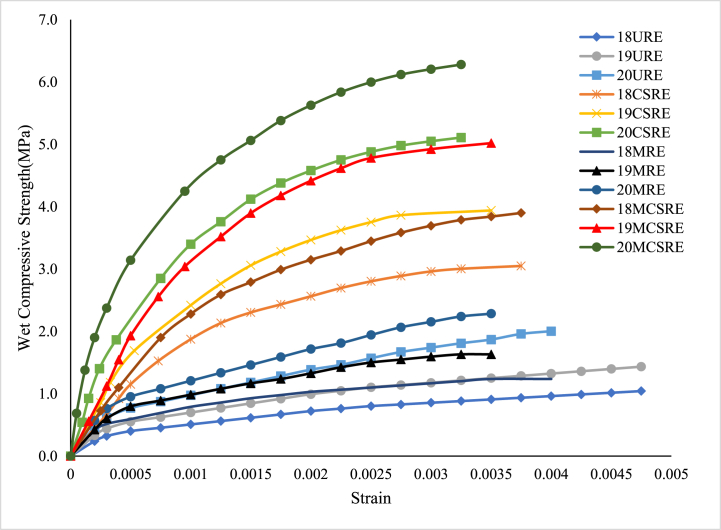
Stress-Strain Curve for URE, CSRE, MRE and MCSRE specimen at 90 day curing.

The stress-strain curve of all rammed earth specimen at a curing period of 90 day is presented in Fig. 6. It was observed that with an increase in the curing period, the compressive strength of RE specimen also increased considerably.

The 90 day wet compressive strengths of URE specimen were in a narrow range of 1.04 MPa–2 MPa for all variations of density. With the stabilization of RE with cement (CSRE), the wet compressive strength increased by 50 % to 3.05 MPa for 18 kN/m^3^, by 100 % to 3.94 MPa for 19 kN/m^3^ and 150 % to 5.11 MPa for 20 kN/m^3^. The average increase in 90 day strength after cement stabilization was 1.7 times when compared to 90 day strength of URE. The percentage gain in strength of URE and CSRE specimen at 90 day is similar to that of previous studies [3,48], where the 90 day strength after cement stabilization increased significantly. The wet compressive strength of MCSRE was significantly higher and was within the range of 3.90 MPa–6.28 MPa for all densities and the resulting increase in strength is within the range of 25 % for all the three densities. The average coefficient of variation of wet compressive strength was 7 %. At 90 day, the strength further improved by 23 % when compared with 28 day strength. The factor that might have effected the strength gain with increasing curing period might be due to the filling of pores by CaCO_3_ produced during the MICP process [17,21]. As the pore space is filled by CaCO_3_, further gain in strength may not be possible. Hence, the improvement in the strength of microbial-induced specimens during the initial stages is more predominant when compared to long-term strength, but is significantly higher in comparison to the long-term strength of CSRE.

The Initial Tangent Modulus (ITM) and the strain at ultimate compressive stress were determined from the stress-strain characteristics and the results are tabulated in Table 3. The 90-day strains at peak strengths are similar to the peak strains of 28 day RE specimens. It is lower for cement stabilized specimens indicating that it has turned relatively brittle. The peak strain for URE was within the range of 0.0040–0.0045 and for MRE was within the range of 0.0035–0.0040. The strain at peak stress for CSRE was within the range of 0.0033–00038 while for MCSRE was within the range of 0.0030–0.0035. The RE specimens without cement stabilization were ductile with higher strain values but with lower stress values, similar to 28 day behavior. However, the specimens stabilized with cement were more brittle with lower strains but with higher strength. The strains were further reduced with MCSRE with a further increase in compressive strength.

**Table 3:** Stress-Strain Characteristics of RE specimens at 90 day curing period.

Dry Density (kN/m^3^)	URE		MRE		CSRE		MCSRE	
	ITM (MPa)	Strain at peak stress	ITM (MPa)	Strain at peak stress	ITM (MPa)	Strain at peak stress	ITM (MPa)	Strain at peak stress
18	1140	0.0045	1890	0.0040	2300	0.0038	4250	0.0033
19	1410	0.0043	2270	0.0035	3060	0.0035	5860	0.0035
20	1970	0.0040	3100	0.0035	3870	0.0033	8900	0.0030

As the curing period increased from 28 to 90 day, the ITM values also increased for all RE specimen. The average ITM increased by 1.3 times as the curing period increased from 28 day to 90 day for all RE specimens. The average ITM values at 90 day increased by 1.9 times with the increase in density from 18 kN/m^3^ to 20 kN/m^3^ for all RE. The ITM increased by two times after cement stabilization. The values are tabulated in Table 3. These findings indicate that the modulus of stabilized RE increases with the induction of bacteria and was also dependent on the curing periods along with variations in density. The results concluded that the compressive strength of microbial induced cement stabilized RE improved with an increase in the curing period, subsequently increasing the ITM.

### 3.4 CaCO_3_ precipitation in RE

**Fig. 7:**
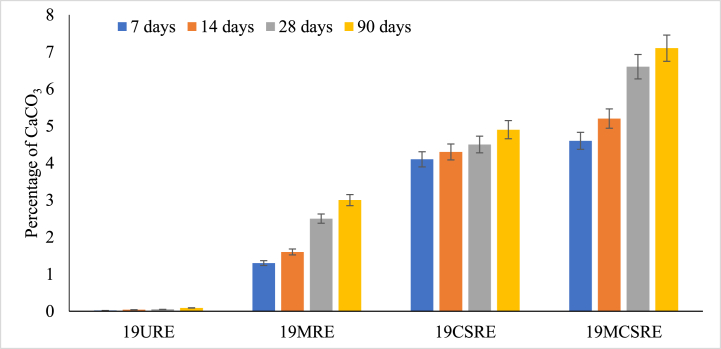
Precipitation of CaCO_3_ in the soil at different periods of curing.

The CaCO_3_ precipitation was determined both in nutrient media and in RE specimen. The weight of the precipitates was 168 mg per 100 ml of NBU media after 4 weeks. CaCO_3_ precipitation of RE samples (of density 19 kN/m^3^) was determined after 7, 14, 28 and 90 day curing periods. The calcium carbonate content (CCC) in unstabilized rammed earth specimen was zero since there were no calcium compounds present in the mix. For microbial-induced unstabilized RE, CCC was found to be 2.5 %. When 19CSRE was induced with microbes, the average CCC increment was 7 % for all curing periods. The percentage of CaCO_3_ formed in the soil at different periods of curing is illustrated in Fig. 7. This increase may be because, calcium carbonate is formed from the combined effects of both cement and microbes.

### 3.5 Capillary water absorption

**Fig. 8:**
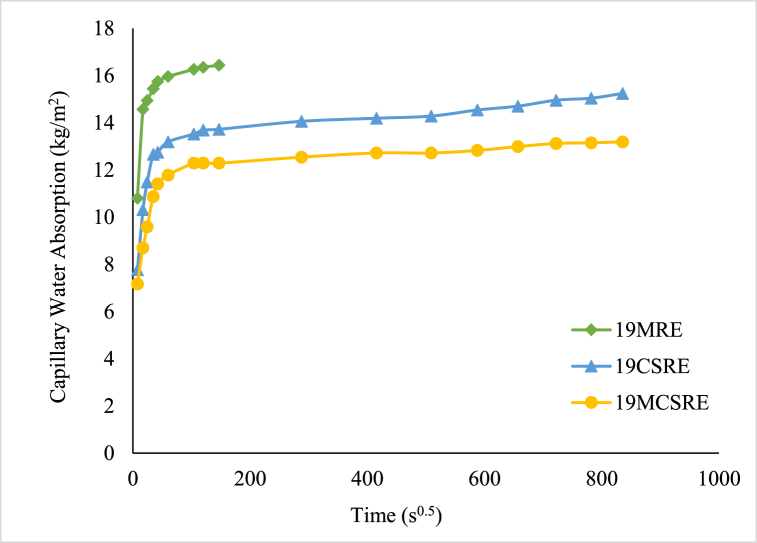
Capillary Water Absorption of RE with respect to time.

The curves in Fig. 8 show the variation of water by capillarity absorption versus the square root of test time for RE specimens of density 19 kN/m^3^. The water absorption results of 19URE samples could not be obtained since they disintegrated when placed in water. The 19MRE samples absorbed water for 6 h and then disintegrated. The capillary water absorption of 19MRE specimens was 16.43 kg/m^2^. The stabilization of RE by adding cement reduces the water absorption rate. Furthermore, the cement-stabilized RE induced with bacteria was less affected by capillarity suction. The 19MCSRE specimens cause lesser porosity in the blocks in comparison to 19CSRE. The capillary water absorption reduced from 15.24 kg/m^2^ to 13.19 kg/m^2^ with the addition of bacteria to cement stabilized RE samples. These results are concordant with those reported for soil blocks [[49], [50], [51], [52]].

**Fig. 9:**
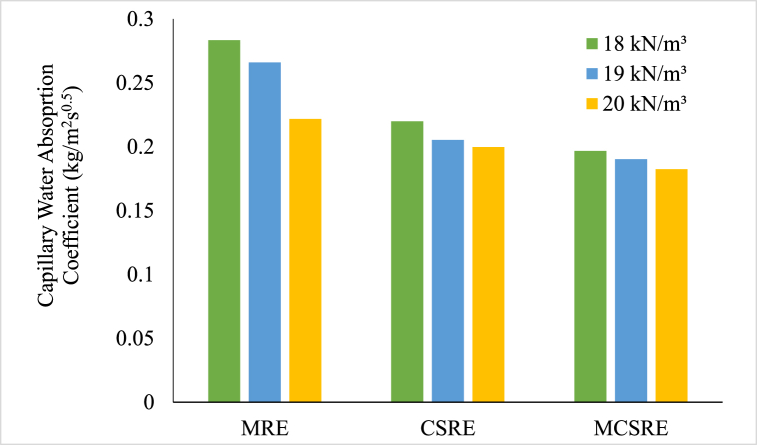
Capillary Water Absorption Co-efficient of RE samples.

The capillary water absorption coefficient is the gradient of the straight portion obtained after plotting the water absorbed per unit area against the square root of time. The capillary water absorption coefficient of all RE samples are shown in Fig. 9. The capillary water absorption coefficient value was at an average of 0.21 kg/m^2^s^0.5^ for CSRE specimen and was 0.19 kg/m^2^s^0.5^ for MCSRE specimen. This result is because of the presence of CaCO_3_ particles that filled the pores and delayed the flocculation of particles contained in the soil which avoids the advancement of cracks and widening of pores. It can be deduced that the decrease in porosity by the precipitation of CaCO_3_ has resulted in an increase in strength and might also reduce the formation of biodegradable matter like fungi in RE.

### 3.6 Correlation between compressive strength and capillary water absorption coefficient

A correlation between the capillary water absorption coefficient and compressive strength of RE specimens can be formulated. The correlation between compressive strength and capillary water absorption coefficient might be essential for understanding the correlation between the strength and water absorption properties of the RE specimens.

**Fig. 10:**
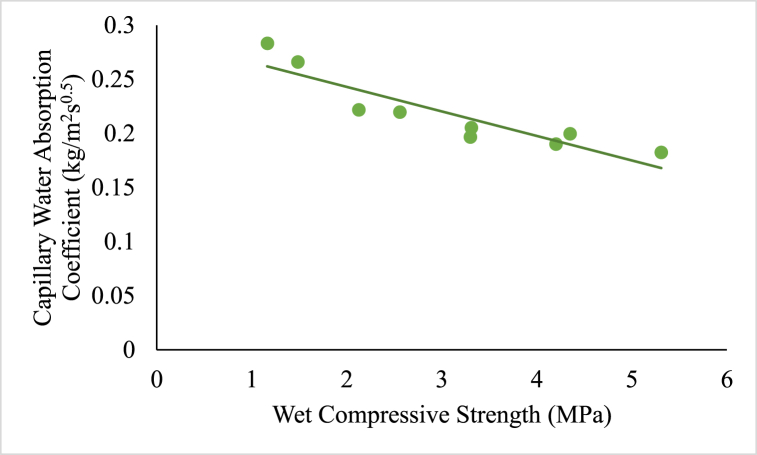
Correlation between compressive strength and capillary water absorption co-efficient of bacteria induced specimens.

A regression analysis of the correlation between compressive strength and capillary water absorption coefficient was performed for RE specimens (except URE). Power regression models were used to find the best equation that describes the experimental data and the equation was assessed to know the best fit using statistical procedures. The relation between compressive strength and capillary water absorption coefficient is linearly declining and is shown in Fig. 10.

The correlation formed for RE specimens with bacteria (MRE and MCSRE) is expressed in Equation (1) as follows:

(1) $$C=-0.024f_{c}+0.29$$

The correlation formed for CSRE specimens is expressed in Equation (2) as follows:

(2) $$C=-0.011f_{c}+0.24$$

where C = Capillary Water Absorption Co-efficient in kg/m^2^s^0.5^

f_c_ = Compressive Strength in MPa.

The strength of the MCSRE specimens improved with a reduction in water absorption. This reduction in water absorption coefficient is credited to the presence of CaCO_3_ particles, which filled the pores, resulting in increased strength. The Pearson correlation coefficient (R) was estimated and is found to be 0.918 for RE specimens with bacteria.

Previous researchers have established similar equations for various cement-based materials have been formulated similarly. Research done on concrete and pozzolanic material defines the correlation as C = −0.024f_c_ + 1.79 [53]. Another research on concrete with waste foundry sand have equated the correlation as C = −0.075f_c_ + 4.49 [54]. Although the values obtained through equations (1), (2) seems to be little approximate given the number of specimens tested, it is an interesting research issue that is worth exploring and more tests are needed to establish a precise formula of this kind.

### 3.7 SEM analysis

In the previous sections of this study, the increment in compressive strength was discussed for RE specimens with and without bacterial induction. This increment in compressive strength can be authenticated through SEM analysis. The behaviour and interaction of bacteria with soil particles and with cement at a micro level are useful to provide substantiation to the observed macro-level changes in the properties of microbial-induced RE specimens as a comparison to the control specimens.

**Fig. 11:**
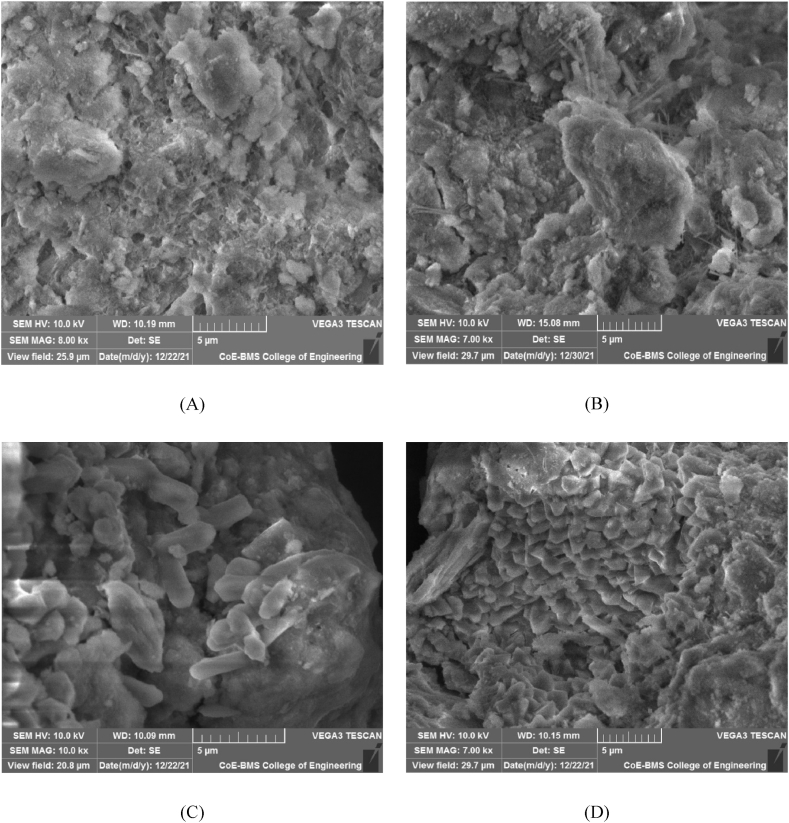
A - Unstabilized RE specimens; **11B** – Control Specimens stabilized with cement; **11C** – Bacterial Cells embedded in RE Specimens; **11D** – Formation of Calcium Carbonate crystals inside RE Specimens.

The SEM images of unstabilized RE specimen showed the occurrence of large voids and did not indicate the formation of strong bonds as shown in Fig. 11A. The SEM images of control specimens stabilized with cement (Fig. 11B) clearly indicated that though a bond was formed due to the hydration of cement forming soil-cement clusters, voids were not filled completely.

**Fig. 12:**
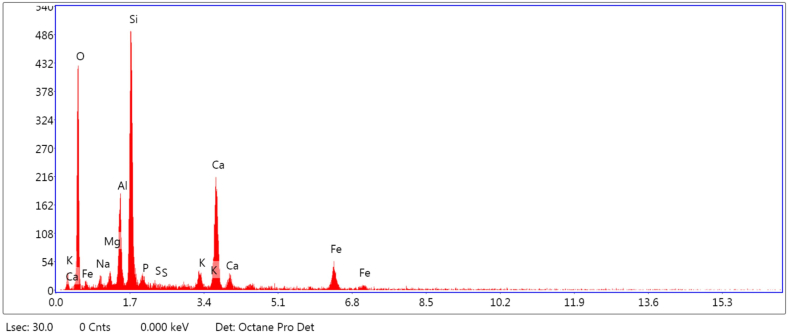
EDX analysis of bacterial-treated RE cylinders.

**Fig. 13:**
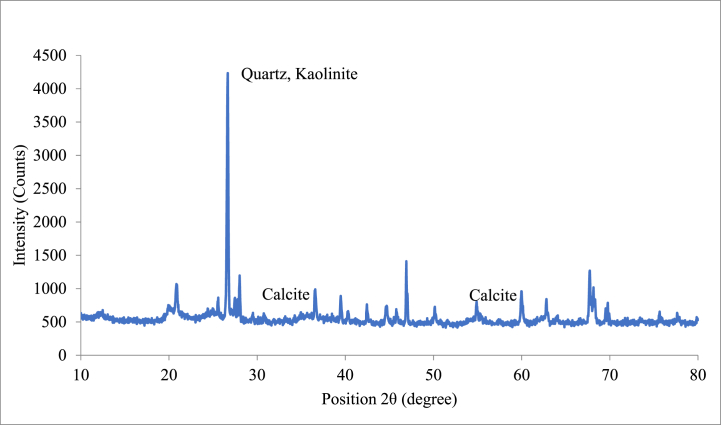
XRD analysis of bacterial-treated RE cylinders.

From Fig. 11C it can be observed that in MICP-treated RE specimen, the bacterial cells were embedded in RE. Embedment of bacterial cells inside the calcium carbonate crystals showed that the void space in the specimens had been occupied by bacteria during crystallisation and the cells were completely occupied by the CaCO_3_ crystals. The SEM images that were captured after 28 and 90 day curing periods of the bacterial specimen evidently showed the deposition of calcium carbonate crystals on soil particles (Fig. 11D). High amounts of calcium in bacteria-treated RE specimens were also established by XRD and EDX analysis (Fig. 12, Fig. 13). The microstructure of RE specimens with MICP treatment was found to be denser and the voids percentage reduced greatly. This dense structure of the soil mass may be credited to the combined effect of cementitious compounds and the compounds formed during the MICP process.

A clear variance can be noted between the microstructure of the RE specimens with and without microbes. These observations show the effectiveness of bacteria in the stabilization of RE along with conventional stabilizers. These micro-level changes also validate the macro-level changes of specimens observed through the wet compressive strength.

## 4 Conclusions

The investigation carried out in this study is aimed at improving the engineering properties of rammed earth by microbial induction. This study has demonstrated the potential of Microbially Induced Calcium Carbonate Precipitation (MICP) as a sustainable and effective method for enhancing the properties of Rammed Earth (RE). The findings confirm that MICP can significantly improve the compressive strength and durability in RE, making it a promising and sustainable construction practice. With MICP, the compressive strength of RE continued to improve significantly beyond 28 day i.e., at 90 day curing period which is usually not the case when cement is used as a stabilizer. It was found that ureolytic bacteria isolates which can adopt well to soil conditions and thrive in alkaline conditions could be effective for the MICP process. Highest cell concentration and peak urease production could be observed after 48 h and 90 h respectively. It was determined that the urease enzyme is the key factor that leads to the precipitation of CaCO_3_ crystals.

The wet compressive strength of cement stabilized rammed earth specimens treated with bacteria showed an average of 26 % higher strength after 28 day of curing when compared with CSRE. The pore space in RE might have been occupied by the CaCO_3_ crystals precipitated by bacteria during 28 day curing period leading to an increase in strength. The 90 day compressive strength of MCSRE specimen increased by 23 % when compared to CSRE, indicating that bacteria have played a role in improving the long-term strength of RE. It was evident that the process of bio cementation due to MICP led to the filling up of pores in MCSRE specimens by precipitating calcium carbonate which consequently resulted in higher compressive strength.

The capillary water absorption of MICP specimen reduced considerably when compared with the untreated specimen with bacterial induction. A linear co relation between compressive strength and capillary water absorption co-efficient is established for MRE, MCSRE and CSRE specimen, according to which a reduction in water absorption increases the compressive strength. Reduction of pores can be termed as a major factor to be considered for the longer life and performance of RE.

From the experimental results of the present study, it can be concluded that the process of bio cementation by MICP to stabilize rammed earth has been effective in increasing the compressive strength by filling up the voids and reducing the capillary water absorption.

## CRediT authorship contribution statement

**Neha Vivek A:** Writing – original draft, Resources, Methodology, Investigation, Data curation. **Prasanna Kumar P:** Writing – review & editing, Supervision, Project administration, Conceptualization. **Divijendranatha Reddy:** Writing – review & editing, Supervision.

## Data availability statement

“The datasets generated and analysed during this study are available from the corresponding author upon reasonable request.”

## Patent disclosure

“The research findings of this research article have been filed for a patent titled “Microbial induction and crack repair in Rammed earth” to protect the novel findings and innovations described herein.” [Patent Application Number: IN202441040272.]

## Ethics in publishing

This research adheres to the ethical guidelines stated in Elsevier's Publishing Ethics Policy. The authors affirm that the study has been conducted in accordance with the highest ethical standards in research and publication.

## Research ethics & compliance

This study does not involve human participants, animals, or sensitive data requiring ethical approval. However, the authors have ensured that all research activities comply with international and institutional ethical standards.

## Authorship & contributions

All authors have significantly contributed to the research, data collection, analysis, and manuscript preparation. The final manuscript has been reviewed and approved by all authors.

## Duplicate publication & related work

This manuscript represents original work and has not been published previously, except in the form of a preprint, abstract, or academic thesis. It is not under consideration for publication elsewhere. The authors confirm compliance with Elsevier's policies on multiple, redundant, or concurrent publication.

## Plagiarism & originality check

The originality of this manuscript has been verified using plagiarism detection tools, in compliance with Elsevier's plagiarism policies.

## Funding

“The authors declare that no funds, grants, or other support were received during the preparation of this manuscript.”

## Declaration of competing interest

The authors declare the following financial interests/personal relationships which may be considered as potential competing interests: Neha Vivek A, Prasanna Kumar P, Divijendranatha Reddy S has patent #IN202441040272 pending to BMS College of Engineering. If there are other authors, they declare that they have no known competing financial interests or personal relationships that could have appeared to influence the work reported in this paper.
